# Why sub-Saharan African health workers migrate to European countries that do not actively recruit: a qualitative study post-migration

**DOI:** 10.3402/gha.v7.24071

**Published:** 2014-05-13

**Authors:** Annelien Poppe, Elena Jirovsky, Claire Blacklock, Pallavi Laxmikanth, Shabir Moosa, Jan De Maeseneer, Ruth Kutalek, Wim Peersman

**Affiliations:** 1 Department of Family Medicine and Primary Health Care, Ghent University, Ghent, Belgium; 2 Unit Ethnomedicine & International Health, Department of General Practice & Family Medicine, Medical University of Vienna, Vienna, Austria; 3 Department of Primary Care Health Sciences, Oxford University, Oxford, UK; 4 Department of Family Medicine, University of Witwatersrand, Johannesburg, South Africa

**Keywords:** migration, brain drain, healthcare, sub-Saharan Africa

## Abstract

**Background:**

Many studies have investigated the migration intentions of sub-Saharan African medical students and health professionals within the context of a legacy of active international recruitment by receiving countries. However, many health workers migrate outside of this recruitment paradigm. This paper aims to explore the reasons for migration of health workers from sub-Saharan Africa to Belgium and Austria; European countries without a history of active recruitment in sub-Saharan Africa.

**Methods:**

Data were collected using semistructured interviews. Twenty-seven health workers were interviewed about their migration experiences. Included participants were born in sub-Saharan Africa, had trained as health workers in sub-Saharan Africa, and were currently living in Belgium or Austria, though not necessarily currently working as a health professional.

**Results:**

Both Austria and Belgium were shown not to be target countries for the health workers, who instead moved there by circumstance, rather than choice. Three principal reasons for migration were reported: 1) educational purposes; 2) political instability or insecurity in their country of origin; and 3) family reunification. In addition, two respondents mentioned medical reasons and, although less explicit, economic factors were also involved in several of the respondents’ decision to migrate.

**Conclusion:**

These results highlight the importance of the broader economic, social, and political context within which migration decisions are made. Training opportunities proved to be an important factor for migration. A further development and upgrade of primary care might help to counter the common desire to specialize and improve domestic training opportunities.

International migration of healthcare workers has sustained the human resources crisis in many countries in sub-Saharan Africa (1, 2). The flow of health professionals from low-income to high-income countries has received much attention over the past few decades and is considered to be a significant contributor to the further weakening of already fragile health systems. The loss of human capital as a result of migration is high. Kasper and Bajunirwe concluded that in half of the countries in sub-Saharan Africa, more than 30% of the physicians trained locally are ‘lost’ as a consequence of migration (3).

Migration processes have become more complex over recent decades (4). This is also true for health worker migration (5). Thirty years ago, health worker migration involved a small number of sending countries in sub-Saharan Africa, and a limited number of high-income countries outside Africa as receiving countries (6). However, Connell et al. (7) report that things have changed significantly since then. Today, nearly all sub-Saharan African countries show increasing outflows of health workers, whilealso the variety of destination countries has largely expanded. Globalization has without doubt played a part in increasing migration flows, but active recruitment policies in high-income countries are also considered an important contributor.

In several African countries, both students as well as health professionals from various disciplines (medicine, nursing, pharmacy, etc.) have been questioned about migration intentions. Different push and pull factors have been identified. Push factors most often cited include lack of opportunity for professional development, unavailability of equipment and supplies, heavy workload, low wages, low job satisfaction, and the threat of political instability and conflict. Pull factors include better remuneration, better working conditions and opportunities for professional development (8–14). In addition to these push and pull factors, Hagopian et al. (13) reported a well-developed culture of medical migration among physicians in sending countries, encouraging them to look for opportunities abroad.

Despite the variety of recipient countries involved, literature is still dominated by research which focuses on migration to the UK, US, and Canada (7). Those countries attract most of the health workers migrating from sub-Saharan Africa, and all have existing or historic active recruitment policies.

During the past decades, high-income countries such as the UK, US, and Canada have filled domestic shortages by the international recruiting of health professionals; many travelling from low-income countries facing critical shortages of health workers. Such active recruitment has been subject to ethical debate and challenge. In response, the WHO developed a voluntary code of practice on the international recruitment of health personnel in 2010, in consultation with all relevant partners. This provided ethical guidelines for the recruitment of international health personnel to protect and strengthen health systems in low-income countries, those in economic transition and small island states (15).

The WHO-code was an important step forward in regulating active international recruitment; however, health worker migration will not cease to exist. It is therefore important to also gain insights into the reasons for migration when there is no active recruitment involved.

Reasons for migration to countries with active international recruitment strategies have received much attention, whilst studies on migration to countries which have never actively recruited sub-Saharan African health workers are lacking. Though this group of migrant health workers is smaller in comparison, they form a silent minority whose voices need to be heard.

Little is known about actual migration experiences and reasons of sub-Saharan African health workers who moved to countries that do not actively recruit from sub-Saharan Africa, such as the case for Austria and Belgium. Although Belgium has no history of active recruitment, it is the eighth most common destination country of African health professionals (16). Austria ranks outside the top 10.

The aim of this study was to explore, with semistructured interviews, the actual (post-migration) reasons of sub-Saharan health workers to migrate to Europe, and more specifically to Austria and Belgium, within a context of no active recruitment.

## Methods

### Design

This study presents data collected through semistructured interviews in two European countries: Belgium and Austria. The decision to use a qualitative approach was based on two aspects: 1) actual reasons for migration of health professionals are relatively unexplored within a context of no active recruitment policies 2) migration experiences and personal stories can be expected to be very diverse. A qualitative approach offers the opportunity to identify the reasons for migration and allows us to acquire views and opinions that cannot be captured using quantitative research methods (17).

### Participants

Participants were selected according to the following inclusion criteria:

1. Born in sub-Saharan Africa.
2. Received professional training in medicine (doctors and medical assistants), nursing, or midwifery in sub-Saharan Africa. Respondents did not necessarily have to have completed their training in sub-Saharan Africa.
3. Living in Belgium or Austria at the time of the interview.

Participants did not have to work as a health professional in Belgium or Austria, because regulations in Belgium or Austria sometimes force migrants to seek work outside their previous professions.

Different recruitment strategies were used to identify potential participants. In both Belgium and Austria, several organizations were contacted: African migrant organizations and federations, organizations undertaking counseling of newly arrived migrants, language centers and organizations active in the process of integration. In Austria, contacts were also made with the Austrian Medical Chamber, the Austrian Nurses Association, and the Vienna branch of the International Organisation of Migration. Organizations that could not provide names or addresses for reasons of confidentiality distributed a flyer to their members or clients, or approached potential participants themselves. Nursing homes and hospitals were asked to distribute flyers and identify potential participants among their personnel. The call for participants was circulated in online fora and flyers were distributed in neighborhoods with a high concentration of African migrants. Finally, a snowballing technique (respondents were asked to name other potential respondents who we could contact (18)) and informal networks were used to identify potential participants. Recruitment strategies were not all equally successful. The majority of the participants were recruited with the help of an intermediary (mainly former respondents). Recruitment through African migrant organizations yielded few participants.

The researchers aimed to compose a heterogeneous sample by age, gender, country of origin, training, and length of residency in Belgium or Austria.

### Data collection

The Department of Family Medicine and Primary Health Care (Ghent University) was responsible for the data collection in Belgium and the Department of General Practice and Family Medicine/Unit Ethnomedicine and International Health (Medical University of Vienna) collected data in Austria. In both countries female researchers (AP and EJ) with experience in qualitative research established contacts with potential participants and conducted the interviews. The participants were asked to suggest a place where the interview could take place. Interviews took place either at the home of the participants, at their own or the researcher's office, or occasionally in a coffeehouse. Those who agreed to participate received an information leaflet before the interview, containing information about the goals and practical aspects of the study. Informed written consent to participation was obtained in each case.

In both countries, the same semistructured topic list was used as guidance for the interview. The determination of the main topics was preceded by a review of available literature on migration motives of sub-Saharan African health professionals. The topics included personal migration motives, migration experience, changes felt necessary in the country of origin to retain health workers, transnational ties, and future plans.

The interview questions were designed to encourage open-ended answers and narrations, as is the aim in semistructured interviews (19). During the interview, the interviewer could freely change the order of questions or add additional themes, depending on the narrative of the participant. Participants were interviewed either in Dutch, French, German, or English. The interviews took between 30 and 90 min. They were recorded and transcribed verbatim by the interviewer (Belgium) or another member of the research team (Austria). Interviews were conducted until thematic saturation (20) was reached, combining Austrian and Belgian data. The research team completed 17 semistructured interviews with migrant health workers in Belgium and 10 in Austria. Interviews were conducted between October 2011 and April 2012.

### Data analysis

The analyses of the interviews were inspired by Grounded theory (21). There was no codebook constructed before. Data were coded using an open coding process. In an iterative process, a label or code was applied to selected text, and codes were clustered, compared and sorted into distinct and comprehensive themes and sub-themes. Themes were compared and sorted in a process of constant comparison (21), in order to refine existing codes and identify new codes (22). For the purpose of this paper, the initial coding of all the interviews and developing of themes was done by AP and EJ. In Belgium, an additional researcher (WP) read through all the transcripts and also independently coded the first interviews. Once reliability was established with one another, AP completed the rest of the coding (22). Code and theme development were compared and discussed, both within and between the two research teams. NVivo software was used for the final coding and analysis of the transcripts.

The Ethics Committee of Ghent University Hospital approved the Belgian study. The Ethics Committee of the Medical University of Vienna approved the Austrian study.

## Results

Twelve men and 15 women were interviewed. The youngest participant was 26 years old and the eldest was aged 59. The participants originated from a wide range of countries in sub-Saharan Africa. Eighteen participants were trained as medical doctors in their country of origin, seven as nurses, and two as medical assistants. Approximately half of our respondents did not work in healthcare at the time of the interview. Most of them were involved in procedures of diploma equivalence, or had already started the necessary extra years of study. Some were unemployed or were working below their level of previous professional achievement, mainly due to problems with bureaucracy and legal issues. The time since they left their home country ranged between 2 months and 34 years. Table 1 provides an overview of the demographic characteristics of the participants.

**Table 1:** Sample characteristics

	*N*=27
Sex	
Male	12
Female	15
Age	
20–30	3
30–40	14
40–50	5
50+	5
Professional background in country of origin	
Physician	18
Nurse	7
Medical assistant	2
Time since they left their country of origin	
− 5 years	8
5–10 years	7
+10 years	12
Country of origin	
DR Congo	4
Rwanda	4
South Africa	4
Guinea	2
Nigeria	2
Angola	1
Burundi	1
Congo Brazzaville	1
Gabon	1
Ghana	1
Ivory Coast	1
Senegal	1
Somalia	1
Sudan	1
Tanzania	1
Uganda	1

The analysis revealed three primary reasons for leaving the country of origin: 1) Educational purposes; 2) Political instability and insecurity; 3) Family reunification. Two respondents mentioned medical reasons as a driver for their migration. Reasons for migration to Austria and Belgium were comparable. There were no remarkable differences between the stories of those who migrated only recently, and those who left their country more than 10 years ago.

### Educational purposes

Many participants, both nurses and doctors, noted that their migration was inspired by the desire to continue their education; seeking to specialize or undertake additional training. When asked why they had such a strong wish to specialize, different reasons arose. First of all, some doctors stated that in their country, the role of a general physician was not really established.

… Because in [home country], the profession of a general physician is not really known. People do not have a general physician. When your child is ill, you take it to a paediatrician or a specialist doctor. Only in rural areas … People don't know that, general physicians. So students always do a specialization …. (respondent 8 – physician – female – Belgium)

It was felt that jobs available for GP's were generally underpaid and mainly situated in deprived rural areas. So if one wanted better conditions, the belief was that one had to orient toward a certain specialty during work, or continue studying. However, in some countries and for some participants the route to specialization was not obvious.

Limited access to specialist training was mentioned several times. Because it was not possible or likely to get accepted, they chose to continue studying elsewhere. The reasons for lack of access to a certain study differed: no appropriate high school training, adjustment of the conditions to get access, political influences.

… Because, afterwards, when you have finished your studies, you can still do a specialization. But there are not many places for that in [home country], they got to choose. And to have that chance, there are also political factors involved. I saw that I would not have a chance. (respondent 2 – physician – female – Belgium)

Before even coming to Europe, one of our respondents, had studied and worked in a North African country:

… In [home country] we had too many people doing their school graduation exam, so we were too many for university. It could not take all students and so we had to use every opportunity to study outside our country. It was almost like a scholarship, and I had my scholarship in [North Africa]. So, I went there to finish my University studies in the medicine college and I also worked there in the hospitals for a while … (respondent 21 – physician – female – Austria)

Furthermore, the difference in perceived quality between European and sub-Saharan African education was mentioned by participants in Belgium. Contrary to a sub-Saharan African degree, they believed that a degree obtained in Europe is internationally recognized and offers more opportunities in the future in sub-Saharan Africa, as well as worldwide. Personal contacts overseas fed this belief.

… I have my granddad who lives in France and it's him who has told me: listen, you are of good will, look for resources to come to Europe, you can study, you can reach a level that's really … a degree that's internationally recognized. For me, my primary motivation was really to study. And he has told me, listen, there, you will really study. Studies which are really … you will have an excellent diploma … (respondent 11 – nurse – male – Belgium)

Another respondent doubted the quality of her sub-Saharan African degree and affirmed that a specialist degree, obtained in Europe, would result in more professional opportunities when she returns home.

… Those who leave for a specialization of 2 or 3 years … when you return, there are more opportunities than when you would have stayed there to practice … Ourselves, we don't have trust in our diploma … (respondent 16 – physician – female – Belgium)

Some just wanted to gain knowledge at a more advanced level and develop professionally. Since there were simply no opportunities to continue with specialist training in their home country, they chose to leave and look for opportunities elsewhere.

… I wanted to do extra special studies … We do not have postgraduate programs in [home country]. I think it's the main reason for a lot of people. They leave for specialties which do not exist in [home country] … (respondent 6 – physician – male – Belgium)

Some health workers cited the pride attached to a degree obtained in Europe.

… Whenever you study in Europe and then go back to Africa you know some kind of pride you know … People look you with one eye, you know, with a kind of respect … (respondent 1 – physician – male – Belgium)

### Political instability and insecurity

A number of respondents left their country for reasons related to political instability, both direct and indirect. Political conflicts in their country of origin involved direct risks to safety for some, forcing them to leave. As one said:

… It is a genocide which is preparing in [home country]. They kill people just like that. Myself, the day I have fled, they tried to kill me. It's that that made me flee. It's to preserve my life … (respondent 7 – physician – male – Belgium)

Furthermore, it was noted that politics affected every aspect of life, including healthcare, which made it difficult to progress professionally.

… But in [home country], it is difficult, because every sector, not only the municipality or … it's politicized everywhere, even in the hospital. It was not easy, because … even the head of the hospital, they are politicized, that's what I want to say … (respondent 2 – physician – female – Belgium)

Someone explained how politics influenced his job performance and made him quit his job as a doctor in a hospital:

… In fact I was on duty. And the person who sat in front of me was a soldier of another ethnic group. So when he saw me, he said: “well, if you don't save my child, I kill you.” It's like that that I fled the hospital …. (respondent 7 – physician – male – Belgium)

All who left because of political risks cited that they felt like the situation in their country was hopeless, and personal safety could not be guaranteed in this sort of context.

… My father and my brother were arrested, they are in jail, we aren't able to live, there is no secure life there. Then I decided to, to go somewhere where I can find better life for us, and yeah, with help of some friends and I, I managed to escape and to come here …. (respondent 21 – physician – female – Austria)

A respondent cited that political instability freezes every possible progress in life:

… If you are in a situation when there is no security, you can't develop your career, you can't … So if you have an opportunity to go in a space where you can walk at your ease, it's obviously … this is the main reason I think for people leaving Africa … (respondent 6 – physician – male – Belgium)

Nearly all of the respondents who left their country for reasons related to political instability and insecurity emphasized that their decision to come to Europe was not a voluntary one and they could not think of any other reason why they would have left. They noted that, if the situation in their country had been politically stable, they would still be living in sub-Saharan Africa.

… Because [home country], it's a beautiful country. When you are working and you do not have problems, I don't really see any reason to leave. Because here, it's not easy at all. No … (respondent 2 – physician – female – Belgium)

… I am here, not because I want to, not because I want the money of the Belgians. It's cold here, in [home country], it's never cold … (respondent 5 – medical assistant – male – Belgium)

Some expressed the wish to return, but they couldn't because of the situation in their country of origin:

… There are so many doctors who are ready to work there freely, but they can't go because of the situation. One of them is me, I can't, I could have gone there and work in [country of origin] and come back, or stay there I don't have any problem. But then apart from that a lot of doctors have been killed in [country of origin]. One of them I was telling you I just started to work in a Pediatric hospital, our director of the hospital was killed! And then there was another one, a Gynecologist was killed again. Last one was one of the best pharmacists, a colleague and a friend of mine and I knew him very well. And he was shot down in front of his clinic … (respondent 19 – physician – male – Austria)

### Family reunification

A number of participants were already personally linked to family or friends in Europe before their migration, and moved within the framework of this relationship. Some met a European partner in their country of origin and they followed him/her to Europe. A doctor cited that for them, staying together in sub-Saharan Africa wasn't an option because of the health risks for their future children:

… Yes, and then it was not an option because we wanted to have children. And children in [home country] is a difficult matter. Because, even when they are very little, a child in [home country] … The risks to die of child illnesses are very high, than for a child in Europe or elsewhere … (respondent 15 – physician – male – Belgium)

Other participants reported that their partner, who originated from the same country, had already left the country earlier and that they had just been waiting for the right time to join them.

The fact that family members already lived in a European country, also made it possible to migrate. Together with the unbearable situation they were often confronted with, this fact very much influenced the decision to leave the home country.

… I had the opportunity to go abroad, because my parents lived in Austria at that time. So this is the reason why I said that when I would come to Europe, I would continue my education and would pursue radiology and not something clinical, that was not interesting for me anymore. It was so much suffering, and no ability to help people … (respondent 24 – physician – male – Austria)

### Medical reasons

Two participants explained that medical issues were involved in their migration decision. One physician reported that her being in Europe was a direct consequence of her disease. Her health status forced her to migrate to Europe, due to the better medical care that can be provided there. She emphasized that, apart from that, she did not have any wish to leave.

… Unfortunately, I developed a pathology, I became sick. It's for that reason that I have come. But I did not come because there are things that … Unfortunately I became sick, I was obliged to come. As a result of that, I am here … (respondent 13 – physician – female – Belgium)

#### Economic factors

Only one participant explicitly stated that he came to Europe because of job opportunities and the prospect of a higher salary. A family member living in Europe informed him about opportunities overseas.

However, in many other cases broader economic factors were also involved in the decision making. For example, a female physician, who came to continue her training and to reunite with her husband, stated that their ultimate goal is to save money and get a qualification that might make life more comfortable when they return.

… But from my side, I think that I will return when I have done an adequate training. Even my husband, he doesn't want to stay here. It's in our search for better living. But when we have a minimum, we are better off in [home country]. With our family, and we can help better there … (respondent 16 – physician – female – Belgium).

Despite primarily leaving because of training and career development, another health worker further reflected on his decision:

… Of course there are some factors surrounding the career, as we are saying … Nobody is saying, I'm leaving because of economic things. But at the end you have your family, your needs, your things … (respondent 6 – physician – male – Belgium)

### Facilitating factors

Many respondents reported that the move to Belgium or Austria was not their first time abroad and many reported a link with Europe, already established before their migration. In most cases this was a personal link, with family members or friends who had already migrated. The Congolese participants that came to Belgium quoted the colonial link between Congo and Belgium. Although not a personal link, they stated that they felt a certain connection and since other specific reasons made them leave their country either way, they felt like Belgium was a logical destination country.

For many others, neither Belgium nor Austria were target countries.

… I didn't plan to come to Austria because if I would like I would prefer to go to somewhere where I can easily learn the language because my colleagues or my friends they are already in London or America …. (respondent 21 – physician – female – Austria)

… I do not know why I have come here. I have arrived in Belgium. But I have never chosen Belgium. They haven't even asked me. There was a friend who helped me. With the help of a smuggler. I wanted to flee, I just wanted to leave my country. It didn't matter which country they sent me to. I even thought that it was somewhere in Africa to where I had left. To my surprise, I saw that it was here. But I did not choose Belgium … (respondent 7 – physician – male – Belgium)

## Discussion

The aim of this study was to understand reasons for the migration (asked post-migration) of sub-Saharan African medical professionals to countries that do not actively recruit. Interviews were conducted in two European migrant–receiving countries, Belgium and Austria, where there has never been active recruitment of health personnel from sub-Saharan Africa.

Although migration was nearly always the outcome of a combination of factors, three principal driving factors were identified: 1) educational purposes; 2) political instability and insecurity; 3) family reunification. Two respondents mentioned medical reasons. Although less explicit, economic factors also played a role in several of the migrants decision to migrate.

The common desire of health workers to specialize, combined with few opportunities to continue studying at a more advanced level, pushed both doctors and nurses toward migration. Many wanted to continue their basic medical training, either because the function of a general physician or nurse is not really known in their country or, because low wages and inferior working conditions and environment define the jobs available to them. Furthermore, political instability and insecurity and family reunification emerged as important factors. Both are unrelated to profession and might also apply to other sub-Saharan African migrants.

Although the primary reasons for migration were not directly related to remuneration, there were often economic factors surrounding the decision. This was the case especially for those who left because of educational purposes. The desire to continue their training was often framed within a search for a better life, either in sub-Saharan African when they return after having obtained the degree, or in Europe.

Strictly job-related factors that have previously been reported as main possible drivers for migration in studies on migration intentions (8–10, 12) were rarely mentioned as principal reasons for migration. The brain drain of health professionals is clearly complex and our interviews emphasized the importance of factors which transcend those at job-level, like political environment and family reunification. Financial factors and working environment did play a role for most of our respondents, but they were not identified as the main drivers and looked upon more as a possible outcome.

It appears that, within a context of no active recruitment, a move to Europe solely for job-related factors like remuneration or job opportunities is not self-evident. This corresponds to the results of the study of Oman, Moulds, and Usher (23), which focuses on the reasons for migration/retention of specialist doctors in Fiji. They concluded that factors like political instability and family welfare predominate for overseas migrants, whilst working conditions or dissatisfaction with career progression could explain the internal brain drain from public to private healthcare.

Globalization, in terms of international experiences and transnational contacts, played an important role in the migration of the interviewed health workers. Previous experiences in foreign countries seemed to have reduced barriers to longer-term migration. Also, our respondents were not pioneers, most of them had personal links overseas that caused or facilitated the move. Participants did not actively choose Austria or Belgium as target destinations. Language barriers and the absence of active recruitment policies could possibly explain this.

At the moment, policy initiatives to address the migration of health workers continue to emphasize the role of financial incentives (24). However, to establish a sustainable result, it is important to look beyond over-simplistic solutions. This study gives rise to a number of other recommendations. If countries want to retain their health workers, providing a secure and safe environment should be first priority. Furthermore, our interviews show that for many health workers, specialist training appears to be a strategy for professional survival. Providing greater job opportunities for general practitioners and nurses might counter the common desire to specialize. The disease-oriented approach, which has predominated healthcare in sub-Saharan Africa, creates a rising demand for and development of specialist doctors instead of generalist doctors. Greater recognition of primary care, including developing Family Medicine as a viable career path for doctors, might be of great value not only for the population, but also in retaining health professionals. However, such an investment needs to be accompanied by efforts to reduce the existing wage gap between generalists and more traditional specialists. Wage gaps have evolved to create value differences between particular specialties, cadres, and work sectors. Not only are specialists paid more than general health workers, but remuneration is also generally higher in private healthcare, and to a lesser extent in non-governmental organizations (NGOs), than in public healthcare, irrespective of training level and the role performed (25). The chances of working in better-paid sectors are far higher with a specialist degree. A reallocation of resources might be required to lift the salary of generalist doctors.

Fur future scientific research, a quantitative study on a large scale would help to better understand the actual reasons for overseas migration, how those reasons are related to recruitment policies and whether they are different from those explaining internal shifts. It would be useful to estimate how many such ‘migrations’ could be avoided, and the cost of doing so (compared to the cost of training more health workers). For example how much more would general physicians need to be paid to remove the desire for specialization? How much would it cost to establish specialist training programs in African countries and how many migrations would these avoid? Political instability is also an avoidable cause but tackling this is probably beyond the powers of health workers, and calculating the cost would be very difficult. Family reunification is perhaps an unavoidable cause of migration and would not be easily changed by any intervention.

By using a qualitative research design, this study brings out a richer and deeper understanding of the complex phenomenon of sub-Saharan African health workers’ decision to migrate, when there is no history of active recruitment. A limitation of the study is that not every respondent was interviewed in his or her first language. However, since all respondents were fluent enough in their chosen languages to clearly answer all the questions, the possible impact this might have on the quality of the interviews is expected to be small. Secondly, approximately half of our respondents did not work in a healthcare setting at the time of the interview. This might have influenced their responses on their reasons for migration.

Unlike Belgium and Austria, other European countries do have a history of recruitment of health workers in sub-Saharan Africa, and since active recruitment policies are considered a main driver of migration (26), results do not apply to all of Europe. Finally, given the small numbers from individual sending countries which are all diverse in both their socioeconomic and political environments, generalizations about sub-Saharan Africa must be interpreted with caution.

## Conclusion

Results show that health workers from sub-Saharan Africa arrived in Belgium and Austria rather by chance, instead of actively choosing this destination for professional reasons. The majority of the moves were facilitated by earlier experiences abroad or transnational contacts. Educational purposes, political instability and insecurity and family reunification emerged as the three principal reasons for migration among health professionals who migrated from sub-Saharan Africa to Belgium or Austria. However, these reasons were not exclusive and decisions for migration were made within a broader social and economic context. Strictly job-related factors like remuneration and working conditions, which are often cited in studies on migration intentions, were rarely mentioned by the participants as direct drivers for their migration. However, although remuneration was not explicitly identified as a driver, economical factors did play a role. For example, in many cases, the desire to continue training was backed by wanting economic progress, either back in sub-Saharan Africa, or in Europe.
